# Nanostructured Oxide-Based Systems for the pH-Triggered Release of Cinnamaldehyde

**DOI:** 10.3390/ma14061536

**Published:** 2021-03-21

**Authors:** Carolina Cionti, Tommaso Taroni, Valentina Sabatini, Daniela Meroni

**Affiliations:** 1 Department of Chemistry, Faculty of Science and Technology, Università degli Studi di Milano, via Golgi 19, 20133 Milan, Italy; carolina.cionti@unimi.it (C.C.); tommaso.taroni@unimi.it (T.T.); valentina.sabatini@unimi.it (V.S.); 2 Consorzio Interuniversitario Nazionale per la Scienza e Tecnologia dei Materiali, via Giusti 9, 50121 Florence, Italy

**Keywords:** controlled release, triggered release, TiO_2_, Schiff base, stability, cinnamic aldehyde

## Abstract

Cinnamaldehyde is a natural product with antibacterial, antifungal, and anti-inflammatory properties, poorly stable in environmental conditions. Systems for the controlled release of cinnamaldehyde are of great interest to the food and pharmaceutical industries. Here, a new oxide-based construct for the release of cinnamaldehyde catalyzed by acidic pH was obtained by a facile grafting method based on amino-silane linkers and imine chemistry. The grafting procedure led to a loading of ca. 5 molecules/nm^2^, determined on oxide powders with CHN and TGA measurements. The covalent grafting of cinnamaldehyde, demonstrated by FTIR analyses, preserved the molecule stability, simplifying storage. Release tests were performed at different pH values (between 5.0 and 7.4). Thanks to imine chemistry, a fast cinnamaldehyde (CIN) release was observed in a pH 5.0 environment. Using 1 mg/mL suspensions, CIN concentrations within the range adopted in the food industry were obtained (12.4 ppm). The grafting procedure was also performed on a porous film based on a photocatalytic oxide, demonstrating the versatility of this method, adaptable to both powders and macroscopic materials. By taking advantage of the photoactivity of the oxide, regeneration of the fouled film was achieved upon UV irradiation for 1 h, opening the door to reusable devices for the controlled release of cinnamaldehyde.

## 1. Introduction

One of the major causes of food spoilage is microbial contamination, which causes the alteration of produce healthiness, nutritional, and sensory features. The consumption of contaminated food can lead to several diseases. The World Health Organization 2015 report estimated the occurrence of about 600 million foodborne illness cases per year and of 420,000 associated deaths [1]. In this context, antimicrobial packaging materials, able to release antimicrobial compounds, are of great interest to the food industry [2,3].

Antimicrobial substances can be synthetic or natural compounds. For the consumers’ safety, most of the research is focused on natural substances [4]. Plant essential oils (EOs) are natural compounds often characterized by antimicrobial activity, which could represent a nontoxic alternative to chemical preservatives in food packaging and storage [1].

Among all EOs, cinnamaldehyde (CIN) is characterized by very appealing properties. CIN is a phenolic terpenoid and the major constituent of cinnamon bark oil; it has been classified as Generally Recognized As Safe (GRAS) by the Food and Drug Administration (FDA) [5]. CIN is a low-cost and highly safe compound, characterized by a characteristic, pleasant aroma [6]. Thanks to its excellent antibacterial [7,8,9], antifungal [10,11,12], anti-inflammatory [13,14,15], and anticancer [16,17,18,19] activity, as well as antioxidant properties [20,21], CIN is widely adopted in the food, cosmetic, biomedical, and pharmaceutical industries [5,22]. For example, in the biomedical field, cinnamaldehyde has been adopted for wound dressing applications [22,23] and in drug delivery systems for cancer treatments [17,18,19,24,25]. However, CIN is characterized by high volatility and poor water solubility, and it can be easily oxidized upon exposure to light, oxygen, and heat [6,22]. These issues limit its antibacterial efficiency and hinder its application in food, agriculture, and other fields [6,22,26]. Recently, the encapsulation of CIN in solid particles or film systems has been proposed to increase its bioavailability and physical stability and decrease its volatility [6,26]. In this respect, most of the literature reports make use of tailored polymer systems for the encapsulation and diffusion-based slow release of CIN [1,5,6,18,19,22,23,26,27,28].

Here, the use of oxide materials, both in powder form and as macroscopic device, for the stabilization and stimuli-controlled release of CIN is reported for the first time. Metal oxides were selected as they are versatile and widespread materials, already found as fillers in polymers [29], as active coatings in food packaging [30,31,32], and as additives in cosmetics [33] and consumer products.

In recent years, research has focused on the possibility to catalyze the release of active molecules through environmental stimuli, such as the matrix pH. The ability to increase the release of CIN by lowering the pH is of great interest to the food and cosmetic industry. Several types of food are characterized by pH values slightly lower than neutrality, such as fresh milk (pH 6.8) [34], black tea (pH 6.7) [35], and vegetables like zucchini (pH 6.5) [36]. On the other hand, it is well known that the growth of several fungi and molds is favored by more acidic pH values [37,38], and that the food spoilage of products involves an initial acidification step [34,39]. For instance, milk is known to acidify until pH values around 5.0 when it ages due to the formation of lactic acid [34].

**Figure 1:**
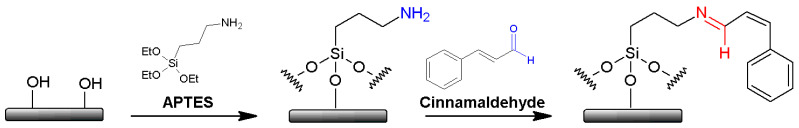
Scheme of the oxide functionalization with APTES and ensuing cinnamaldehyde grafting reaction.

In this context, oxide particles and films can support the pH-controlled release of CIN. This study presents a simple CIN-grafting procedure based on imine chemistry and on alkylsilane linkers. Oxides can be easily functionalized with alkylsilanes in order to modify their surface properties [40,41]. Here, (3-aminopropyl)triethoxysilane (APTES) was adopted for the surface functionalization of the oxide surface; the terminal amine group of the silane (-NH_2_) could then be used for a condensation reaction with the aldehydic group of CIN (-HC=O), yielding an imine bond (-HC=N-) between APTES and CIN (Figure 1). The imine bond is known to be sensitive to the pH of the environment, and its hydrolysis is catalyzed at low pH values. Release tests were thus carried out between pH 5.0 and 7.4, thus within a range that includes the characteristic values of numerous types of food [34,35,36,42,43,44,45].

The grafting procedure here reported can be applied to different oxides and substrates. In this study, the functionalization of both powders and macroscopic porous films was demonstrated. The latter are particularly interesting in view of reusable devices. In this respect, if a semiconductor photocatalyst, such as ZnO or TiO_2_, is adopted as an oxide substrate, its self-cleaning properties can be exploited to regenerate the used surface. In this study, TiO_2_ was adopted as oxide substrate and its photocatalytic activity was used to regenerate the used and purposefully fouled surface, opening the door to multiple reusage of the device.

## 2. Materials and Methods

Reagents were purchased from Merck (Darmstadt, Germany), unless specified otherwise. Milli-Q water was adopted for the preparation of all solutions and suspensions.

### 2.1. Particles Functionalization with APTES

For the preparation of the functionalized powder, a commercial TiO_2_ powder (P25, Evonik, Germany) was adopted as substrate. Figure 1 reports the scheme of the functionalization process. Functionalization with APTES was carried out in a jacketed reactor at 65 °C in N_2_ atmosphere: 500 mg of TiO_2_ powder was added to 50 mL of anhydrous toluene, and the suspension was magnetically stirred for 1 h. Then, 1 mL of APTES was added, and the reaction mixture was stirred for 3 h at 65 °C and in N_2_ atmosphere. The functionalized powder was then collected by centrifugation, washed four times with toluene, and dried in a ventilated oven at 70 °C for 24 h. The obtained sample was labeled as TO-APTES.

### 2.2. Cinnamaldehyde Grafting on APTES-Functionalized Particles

Figure 1 also reports the scheme of the grafting process with CIN. For the grafting of cinnamaldehyde onto the functionalized particles, 400 mg of TO-APTES was dried in a ventilated oven overnight before the reaction to remove any physisorbed water. The reaction was carried out in a jacketed reactor at 65 °C equipped with a reflux condenser and a drying CaCl_2_ tube. While bubbling N_2_, 400 mg of TO-APTES was added to 50 mL of anhydrous methanol. The suspension was magnetically stirred for 1 h, then 201 µL of cinnamaldehyde was added. After 3 h of stirring at 65 °C in N_2_ atmosphere, the product was collected by centrifugation, washed five times with methanol, and dried in a ventilated oven at 70 °C for 24 h. The obtained sample was labeled as TO-APTES-CIN.

### 2.3. Porous Films’ Preparation

Titania porous films were prepared using a template method starting from a titania nonaqueous sol and using polystyrene nanoparticles (NPs) as hard templates (as shown in Figure S1). Polystyrene NPs were prepared by adapting a synthetic procedure from the literature [46] (Section S1). The titania sol was prepared according to a previously reported procedure [47]. Briefly, 126.24 g of ethanol, 27.28 g titanium isopropoxide, 1 mL of 37% aqueous HCl solution, and a previously prepared solution of 0.47 g of Lutensol ON 70 (BASF, Ludwigshafen, Germany) in 102.57 g of ethanol were mixed for 1 h at room temperature under magnetic stirring. To ensure compatibility with the nonaqueous titania sol, water-suspended polystyrene NPs (60 µL) were mixed with particles suspended in ethylene glycol (240 µL). Then, 500 µL of titania sol was added to the mixture and deposited on a 2 × 2 cm^2^ Pyrex glass substrate. The glass slides were previously cleaned by sonication in isopropanol, acetone, and water then immersed in a concentrated H_2_SO_4_ solution and rinsed with water. The film deposition was performed by spin coating, in a single layer, using a spinning rate of 1500 rpm for 20 s, with an acceleration of 500 rpm/s. Films were then calcined at 400 °C (3 h warming ramp and 1 h stationary) under O_2_ flux to remove the NPs and to promote the TiO_2_ crystallization.

### 2.4. APTES Films’ Functionalization and Cinnamaldehyde Grafting

Films were functionalized with APTES using an experimental procedure similar to the one reported in Section 2.1. The reaction was carried out in 20 mL of anhydrous toluene, and 400 µL of APTES was added.

CIN grafting was performed in 20 mL of anhydrous methanol using 80 µL of cinnamaldehyde, according to the procedure reported in Section 2.2.

### 2.5. Material Characterization

Fourier-transform infrared (FTIR) spectra were obtained on a Spectrum 100 spectrophotometer (PerkinElmer, Waltham, MA, USA) attenuated total reflection (ATR) mode using a resolution of 4.0 and 256 scans, in a range of wavenumber between 4000 and 400 cm^−1^. A single-bounce diamond crystal was used with an incidence angle of 45°.

ζ-potential measurements were performed with a Zetasizer Nano instrument (Malvern Panalytical, Malvern, UK). Samples were prepared by suspending the powders (0.5 mg mL^−1^ suspensions) in a 10^−2^ M KNO_3_ aqueous solution. The isoelectric point was determined by adjusting the pH with HNO_3_ and KOH 10^−3^ M solutions.

Elemental CHN analyses were carried out on a CHN 2400 analyzer (PerkinElmer, Waltham, MA, USA).

A TGA/DSC 3+ instrument (Mettler Toledo, Columbus, OH, USA) equipped with a 70 μL alumina crucible was used to perform thermogravimetric analyses (TGA). Measurements were carried out in a 30–900 °C temperature range at a 5 °C min^−1^ warming rate and in air atmosphere.

UV–vis spectra were collected on a Shimadzu UV-2600 (Thermo Fisher, Waltham, MA, USA) between 200 and 800 nm.

A Hitachi TM1000 instrument (Hitachi, Tokyo, Japan) operating at 15 kV was used for the acquisition of scanning electron microscopy (SEM) images.

The polystyrene particle size was evaluated via transmission electron microscopy (TEM) and dynamic light scattering (DLS). Latex samples were deposited onto a 300-mesh copper grid and examined by an EFTEM Leo912ab TEM (Zeiss, Jena, Germany) operating at 80 kV. Digital images were acquired by an Esivision CCD-BM/1K system. DLS measurements were performed with a Zetasizer Nano instrument (Malvern Panalytical, Malvern, UK) by diluting the latex in Milli-Q water (0.5 mg mL^−1^ suspensions).

### 2.6. Release Tests

Release tests were carried out in several water matrices at different pH values. Both the grafted powders and films were tested in phosphate buffer solution (pH 7.4), while tests in acidic pH were performed in citrate buffer (pH 5.0) for grafted powders and in ultrapure water (pH 5.5) for the grafted films. The preparation of the buffer solutions is reported in Section S2. 

Tests were performed by suspending 25 mg of the grafted powder in 25 mL of aqueous solution. The used batch reactor was kept in the dark at a controlled temperature (25 °C) while magnetically stirring. At chosen time intervals, the suspension was sampled, and the powder was removed by centrifugation before measuring the absorption at 285 nm of released cinnamaldehyde by UV–vis spectroscopy [48,49,50,51]. The relative calibration curve is reported in Figure S2. In the case of porous films, a single substrate was immersed in 5 mL of solution. The same protocol for sampling and determination of released cinnamaldehyde was used.

Additionally, selected samples were analyzed by liquid chromatography–mass spectrometry (LC-MS). Analyses were performed on a LCQ Fleet ion trap mass spectrometer (Thermo Fisher, Waltham, MA, USA) equipped with a UPLC UltiMate 3000 system and a UV detector. The adopted column was Zorbax RX-C18 2.1 × 150 mm, 5 μm (Agilent Technologies, Santa Clara, CA, USA). The column oven was kept at 30 °C. A gradient elution at a 0.25 mL min^−1^ flow rate was used for chromatographic separations. Mobile phases were composed of 0.1% formic acid in water (Solvent 1) and 0.1% formic acid in acetonitrile (Solvent 2). A 25 min run time was set with gradient elution: 0.0–5 min (30%, Solvent 1), 5–25 min (100%, Solvent 2). An injection volume of 5 μL was adopted, and the selected detection wavelength was 285 nm. The mass spectrometer was performed with electrospray ionization in the positive ion mode. Full-scan mass spectra were recorded in the mass/charge (m/z) range of 50–1000.

### 2.7. Regeneration Tests

Regeneration tests were carried out on film substrates by purposely fouling their surface with long alkyl chain moieties, followed by regeneration by UV light irradiation. Film fouling was carried out by the chemical vapor deposition of 100 µL of triethoxy(octyl)silane, performed according to a previously reported procedure [47]. Films were then UV-irradiated with a HG500 lamp (Jelosil, Vimodrone, Italy) with effective irradiation power of 30 mW cm^−2^. Fouling and surface regeneration were monitored via static contact angle measurements, performed on a Easydrop instrument (Krüss, Hamburg, Germany) using ultrapure water droplets of 8 µL.

## 3. Results and Discussion

### 3.1. Powder Substrates

The TO-APTES and TO-APTES-CIN powders were characterized in depth in order to confirm the formation of the imine bond (C=N) and to quantify the functionalization and grafting degree. Pristine oxide (named TO in the following) was also characterized as a reference.

Figure S3 reports the ζ-potential curves of the TO and TO-APTES powders as a function of solution pH. The isoelectric point (iep) of the TO reference is around pH 5.9, in agreement with literature reports [52]. Upon APTES functionalization, a shift of the iep to higher pH values is observed (iep of TO-APTES at pH 8.6), in good agreement with the literature on APTES-modified oxides [53,54]. This result supports the presence of free -NH_2_ terminal groups attached to the oxide surface, as further supported by FTIR analyses (vide infra).

**Figure 2:**
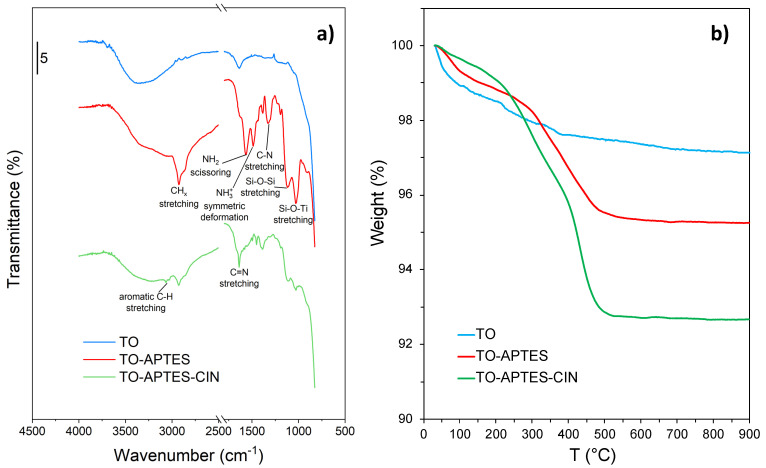
(**a**) FTIR spectra and (**b**) TG analyses of TO, TO-APTES, and TO-APTES-CIN.

The success of surface functionalization with APTES is also confirmed by FTIR spectra (Figure 2a). Compared to the bare oxide, the TO-APTES spectrum presents characteristic bands ascribable to APTES [40]: CH_x_ stretching at 2950–2850 cm^−1^, NH_2_ scissoring and symmetric -NH_3_^+^ deformation at 1567 and 1487 cm^−1^, respectively, C-N stretching mode at ca. 1330 cm^−1^, and Si-O-Si and Si-O-Ti stretching modes at 1120 and 1030 cm^−1^. In the TO-APTES-CIN sample, additional bands are observed in the 3070–3030 cm^−1^ range (related to aromatic C-H stretching) and in the 1650–1300 cm^−1^ region, which can be attributed to the presence of cinnamaldehyde, as supported by comparison with the cinnamaldehyde FTIR spectrum (Figure S4). In this respect, it should be noted that the sharp band at 1635 cm^−1^ is instead absent from the spectrum of pure cinnamaldehyde and can be attributed to the formation of the C=N bond. A further confirmation of the formation of this bond comes from the absence in the TO-APTES-CIN spectrum of the aldehyde C=O stretch, which in pure cinnamaldehyde appears as an intense band at ca. 1680 cm^−1^.

The functionalization and grafting surface densities of TO-APTES and TO-APTES-CIN were calculated on the basis of TGA and CHN measurements.

TGA curves are reported in Figure 2b. The TO reference sample shows a weight loss of about 3%, the majority of which occurs under 200 °C and can be correlated to the loss of the oxide surface hydration. The functionalized TO-APTES powder presents a similar behavior to bare TO at low temperatures, showing, however, a lower degree of surface hydration. At higher temperatures, between 280 and 450 °C, the TG curve of TO-APTES is characterized by a marked weight loss (around 3%). This weight loss can be attributed to the degradation of the alkyl chains of APTES molecules. The TO-APTES-CIN sample displays a similar behavior to TO-APTES up to ca. 400 °C, showing even lower surface hydration below 200 °C and a 3% weight loss between 280 and 400 °C. At higher temperatures, the CIN-grafted particles show a further drop in weight (3%) up to ca. 500 °C, possibly due to the thermal degradation of the cinnamaldehyde aromatic ring. It is thus possible to estimate the functionalization degree of APTES in TO-APTES and in TO-APTES-CIN, and the grafting surface density of CIN in TO-APTES-CIN. All the relative calculations are reported in Section S3.1. The results suggest a surface density of APTES residues, δ_APTES_, in TO-APTES and TO-APTES-CIN of 6.9 and 3.5 molecules/nm^2^, respectively. The observed decrease in the APTES surface density in TO-APTES-CIN can be ascribed to a loss of physisorbed APTES molecules during CIN grafting in methanol. The value of 3.5 molecules/nm^2^ is compatible with a complete monolayer of APTES molecules at the oxide surface and is in good agreement with previous reports [40]. The surface loading calculated for CIN molecules is 5.1 molecules/nm^2^, highlighting a small excess of CIN molecules with respect to APTES moieties. This can be explained with the presence of a fraction of physisorbed CIN along with covalently grafted CIN molecules.

The present results are in good agreement with CHN results reported in Table 1.

Table 1 shows that the C/N molar ratio of TO-APTES is 3.4, which is very close to the theoretical value for an APTES residue that has lost all three ethoxy groups. Therefore, an almost complete hydrolysis of the ethoxy moieties can be hypothesized. With this assumption, the theoretical C/N molar ratio for covalently bonded APTES-CIN moieties (C_12_H_16_NSiO_3_) equals 12. The experimental value for the TO-APTES-CIN sample is slightly larger than the theoretical one: mol_C_/mol_N_ = 14.9. These results suggest that nearly all APTES amine groups reacted to cinnamaldehyde molecules, and that a small amount of physisorbed CIN molecules is present, in good agreement with TGA findings.

The surface densities of APTES and CIN moieties were also calculated from CHN measurements; the relative calculations are reported in Section S3.2. The calculated surface density of APTES moieties in TO-APTES was 10.4 molecules/nm^2^, which corresponds to an APTES multilayer [55]. Although this value is higher than the one obtained by TGA, both techniques support a multilayer coverage for TO-APTES. Conversely, δ_APTES_ determined by CHN in the TO-APTES-CIN sample was 4.0 molecules/nm^2^, a value compatible with a complete APTES monolayer [40]. As in TG analyses, a loss of physisorbed APTES molecules was observed upon CIN grafting. The surface density of CIN in TO-APTES-CIN powders was instead estimated to be 5.3 molecules/nm^2^, which closely mirrors the value obtained from TGA. A small excess of CIN with respect to APTES molecules is consistently observed in TO-APTES-CIN, probably due to the presence of some physisorbed aldehyde.

Release tests were carried out by suspending the TO-APTES-CIN powders for 24 h in solutions at different pH values. HPLC-MS analyses were performed on both a freshly prepared reference cinnamaldehyde solution and on a solution after release test in ultrapure water (Figure S5). Both chromatograms show a main peak at 6.4 min, which can be attributed to CIN according to ESI-MS spectra (m/z 133, [M+H]^+^). Minor impurities are present in both the reference solution and the release test solution, being more appreciable in the latter.

**Figure 3:**
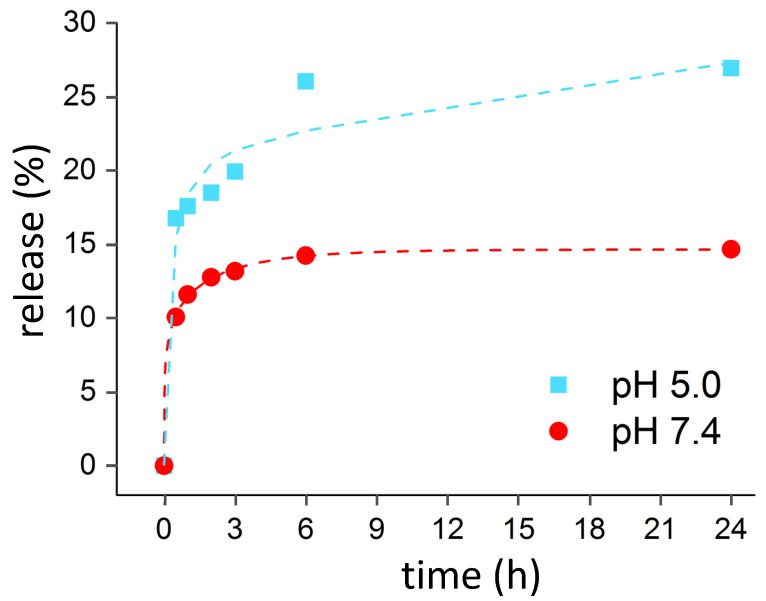
Cumulative release tests of TO-APTES-CIN powder at different pH values. Lines are added only as a guide for the eye.

Since tests in ultrapure water (data not shown) showed a significant lowering of the solution pH, possibly due to alterations of the chemical equilibria of the solution by the released compound and the oxide powder, tests with TO-APTES-CIN powders were carried out in buffer solutions (pH 7.4 and 5.0)** to more rigorously control the pH value. Figure 3 reports the cumulative release curves of CIN for the tests performed at pH 5.0 and 7.4. In both tests, the maximum concentration of CIN was reached within 24 h, but the release test at pH 5.0 is characterized by a much higher CIN release with respect to the one performed at pH 7.4. While the CIN released at pH 7.4 seems to be related to physisorbed molecules, the higher amount of CIN released in acidic conditions supports the pH-triggered release of imine-bonded CIN molecules. Remarkably, the concentration of CIN at pH 5.0 reached a value of 12.4 ppm, which lies in the concentration range of CIN adopted in the food industry to avoid the proliferation of bacteria and fungi [27,28]. In this respect, it should be noted that fresh milk has a pH of 6.8, which acidifies until values around pH 5.0 when it becomes sour [34]. To mimic these real-world conditions, a released test was performed with a pH 6.8 buffer solution. Figure S6 shows that the release curve at pH 6.8 mirrors that at pH 7.4. Stronger acidic conditions (i.e., pH around 5) are needed to heighten the release of CIN, making the system potentially suitable for application in milk matrices.

**Figure 4:**
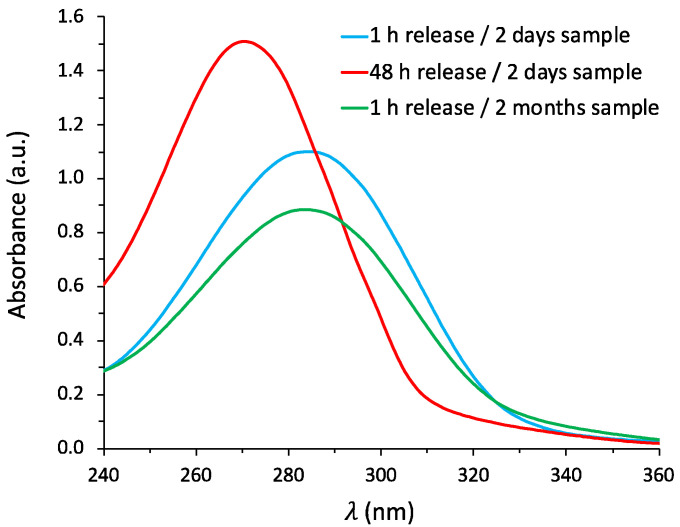
UV–vis spectra of released CIN during tests performed on TO-APTES-CIN after 2 days and 2 months of aging. The spectrum of released CIN after 48 h is also reported as a reference.

It should be noted that CIN is poorly stable once in aqueous solution. Degradation of the CIN in solution becomes apparent after only 48 h, as shown by the clear blue shift in the UV–vis absorption peak reported in Figure 4 (red line). In this respect, grafting via an imine bond could stabilize the CIN molecules against environmental degradation by protecting the labile aldehyde functional group, which is prone to oxidation in environmental conditions [6,22]. To evaluate this aspect, release tests were repeated after several weeks from the synthesis; samples were stored at ambient temperature and exposed to ambient air. Figure 4 compares the UV–vis spectra of solutions from release tests performed on samples with different aging (two days vs. two months). The two spectra (green and blue lines) show no clear difference in terms of absorption wavelength and intensity and are comparable to the spectrum of the as-prepared CIN aqueous solution, demonstrating the increased stability of CIN.

### 3.2. Macroscopic Device

While the use of powder substrates is convenient for formulation and composite preparation, macroscopic substrates are preferable when reusable devices are needed. Here, porous titania films were adopted as macroscopic reusable substrates to maximize loading and to endow self-cleaning performance that could be used to regenerate the oxide surface after usage. Films were prepared by a template route using polystyrene NPs with a size of 381 ± 20 nm and 364 ± 4 nm for NPs synthesized in ethylene glycol and water, respectively (Figure S7). The obtained films presented a homogeneous porosity of around 300 nm in size (Figure S8), as expected on the grounds of the polystyrene particle size and pore shrinkage due to the oxide crystallization.

**Figure 5:**
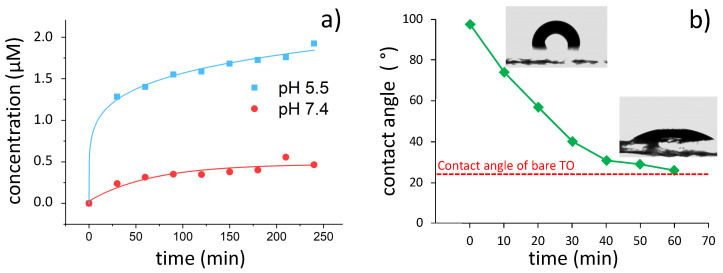
(**a**) Release test with the grafted film at different pH values. (**b**) Regeneration test of the porous film with images of contact angle values (inset); the contact angle of the pristine titania film (red line) is indicated as a reference. In (**a**,**b**) lines were added as a guide for the eyes.

Figure 5a reports the cumulative release of an individual porous film functionalized with APTES-CIN, in solutions at different pH values. In this case, the test in ultrapure water did not result in any appreciable alteration of the solution pH, possibly due to the lower available oxide surface area and lower final CIN concentrations. Thus, for film release tests, ultrapure water (pH 5.5) was adopted as an alternative to the buffer at pH 5.0 to mimic a more realistic scenario for food applications. As in release experiments with powders, acidic pH values lead to a markedly higher concentration of released CIN with respect to pH 7.4.

By adopting a photocatalyst as oxide support, its self-cleaning properties can be used to restore the oxide surface after use by UV irradiation [56]. The self-cleaning properties of the films were tested by purposely fouling a titania film with long alkyl chain moieties and monitoring their degradation under UV light irradiation via water contact angle measurements (Figure 5b). The contact angle (θ) of the fouled surface becomes hydrophobic (θ = 98°) due to the presence of the hydrocarbon chains. Light irradiation activates the photocatalytic degradation of the alkyl chains, leading to a rapid decrease of the contact angle. After 60 min, the contact angle drops to values fully comparable to the clean, pristine surface, demonstrating a fast regeneration of the film surface. This performance was also demonstrated on functionalized films, which were regenerated after usage by light irradiation and later re-functionalized, displaying the reusability of the device.

## 4. Conclusions

In this work, a facile approach, based on oxide-based hybrids, for the storage and pH-triggered release of cinnamaldehyde was reported. The adopted grafting procedure, based on alkylsilane linkers, is easily adaptable to a variety of oxide substrates, both in powder form and macroscopic devices, depending on the desired application. Cinnamaldehyde is linked via its labile aldehyde functional group, which is indeed protected by the grafting procedure, simplifying the long-term storage of this sensitive compound in environmental conditions. Surface density values of APTES and CIN moieties support the formation of a dense APTES monolayer in CIN-functionalized powders, with a slightly higher surface density of CIN moieties. In this respect, it should be noted that two independent determinations (TGA and CHN analyses) provided fully comparable results. These results, along with spectroscopic characterization and release tests at controlled pH, suggest that surface functionalization occurs mainly via covalent grafting by an imine bond, with a minor fraction of physisorbed cinnamaldehyde. In aqueous medium, a pH-triggered release of CIN was observed, at concentrations within the range adopted in the food industry to avoid the proliferation of bacteria and fungi. Further studies are needed to determine the release kinetics in real matrices, such as food models, and to demonstrate the antimicrobial activity of the hybrid material. By employing a photocatalyst as oxide support, a self-cleaning system was developed, which could be easily regenerated after use by UV irradiation for further functionalization and reuse.

## Figures and Tables

**Table 1:** Elemental analysis (in weight percentage) of TO, TO-APTES, and TO-APTES-CIN by CHN measurements.

Sample	%C	%H	%N
TO	0.32	0.56	0.06
TO-APTES	3.23	1.30	1.11
TO-APTES-CIN	5.50	0.64	0.43

## Data Availability

The data presented in this study are available on request from the corresponding author.

## Supplementary Materials

The following are available online at https://www.mdpi.com/1996-1944/14/6/1536/s1, Figure S1: Scheme of the porous film preparation; Section S1: Synthesis of polystyrene NPs; Section S2: Preparation of buffer solutions; Figure S2: calibration curve of CIN; Figure S3: ζ-potential curves of bare TO and TO-APTES powders; Figure S4: FTIR spectrum of cinnamaldehyde; Section S3: Determination of surface densities of APTES and CIN; Figure S5: powder release tests at pH 6.8 and 7.4; Figure S6: HPLC-MS measurements of a CIN reference solution and of a solution after release test; Figure S7: TEM image of polystyrene NPs in water and DLS size distribution by the number of polystyrene NPs in ethylene glycol; Figure S8: SEM image of the porous titania film.
