# Supplementary material for: Nanostructured Oxide-Based Systems for the pH-Triggered Release of Cinnamaldehyde
Source: Materials (Basel). 2021 Mar 21;14(6):1536. doi: 10.3390/ma14061536 (PMC8003980; doi:10.3390/ma14061536)
Supplement: Supplementary file 1 [file materials-14-01536-s001.pdf]

materials

MDPI

Supplementary material

Nanostructured Oxide-Based Systems for the pH-Triggered Release of Cinnamaldehyde

Carolina Cionti [1,2], Tommaso Taroni [1,2], Valentina Sabatini [1] and Daniela Meroni [1,2,*]

[1] Department of Chemistry, Faculty of Science and Technology, Università degli Studi di Milano, via Golgi 19, 20133 Milan, Italy; carolina.cionti@unimi.it (C.C.); tommaso.taroni@unimi.it (T.T.); valentina.sabatini@unimi.it (V.S.)

[2] Consorzio Interuniversitario Nazionale per la Scienza e Tecnologia dei Materiali, via Giusti 9, 50121 Florence, Italy

* Correspondence: daniela.meroni@unimi.it; Tel.:+39-0250-314-220

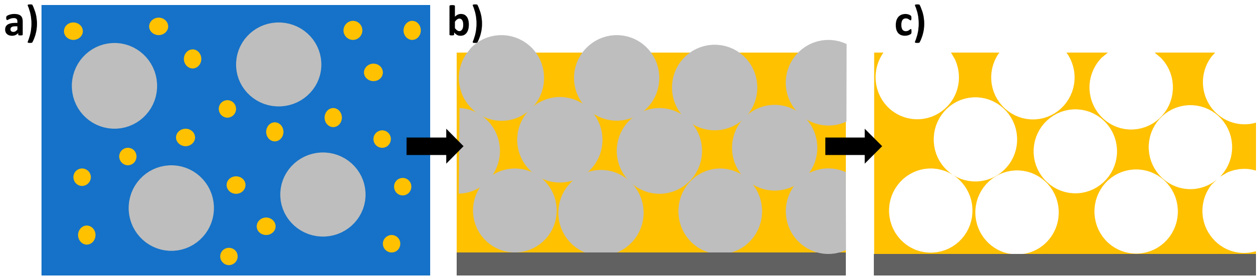

Figure S1. Scheme of the porous film preparation: (**a**) polystyrene NPs (gray spheres) suspension in the titania sol; (**b**) film after spin-coating deposition; (**c**) porous film after calcination at 400 °C under O_2 flux to remove the NPs.

S1. Synthesis of Polystyrene NPs

Polystyrene latices were synthesized by adopting a procedure reported by Goodwin et al. [1] for the preparation of water-based monodisperse polystyrene latices. Briefly, 0.29 mM $K_2S_2O_8$ was dissolved in 100 mL of the chosen solvent (either water or ethylene glycol) in a 500 mL two-neck round-bottom flask equipped with a water-cooled reflux condenser, under nitrogen atmosphere and magnetic stirring. The flask was immerged in an oil bath, the stirrer was adjusted to 400 rpm and then styrene (0.57 M) was added drop-wise. The polymerization reaction was heated for 24 h at 95 °C and then gradually cooled to room temperature. The obtained latices had a final concentration of 3.9%w in water and 5.7%w in ethylene glycol.

S2. Buffer Solutions

The citrate pH 5.0 buffer and phosphate pH 7.4 buffer solutions were prepared according to a procedure reported in the literature [2,3]. The citrate buffer at pH 5.0 was prepared by mixing 20.5 mL of a 0.1 M citric acid aqueous solution with 29.5 mL of a 0.1 M sodium citrate aqueous solution, then diluted to 100 mL. The phosphate buffer at pH 7.4 was instead prepared by mixing 19 mL of a 0.2 M KH_2PO_4 aqueous solution with 81 mL of a 0.2 M Na_2HPO_4 aqueous solution, then diluted to 200 mL.

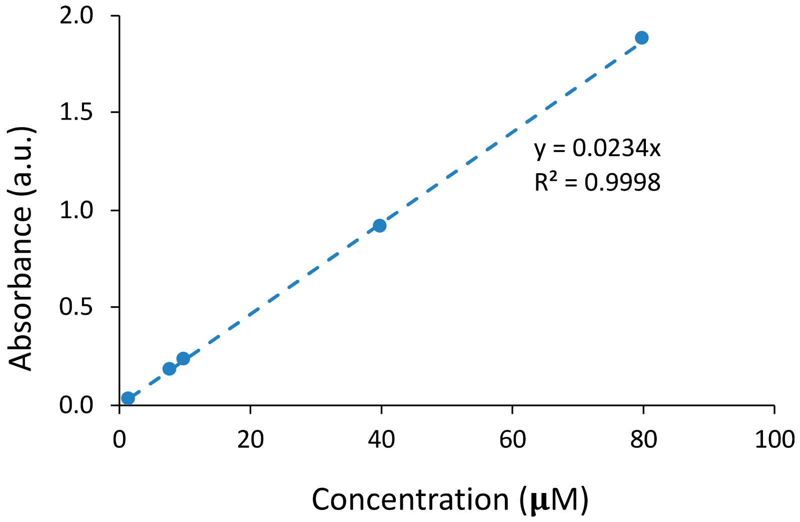

Figure S2. Calibration curve of CIN.

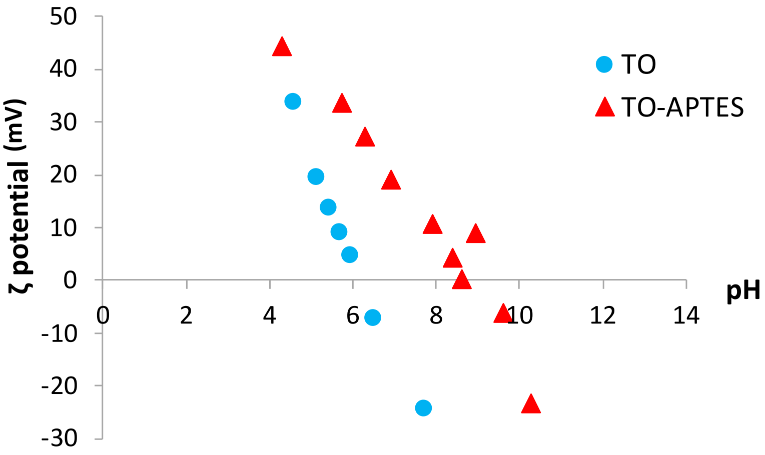

Figure S3. ζ-potential curves of bare TO and TO-APTES powders.

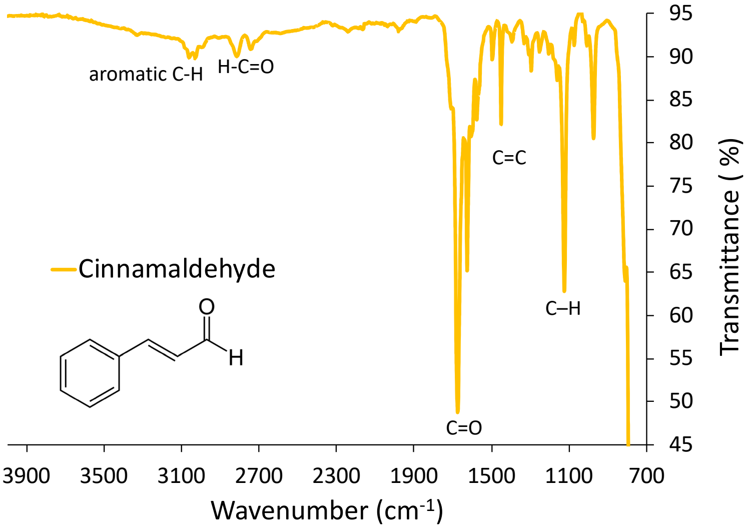

Figure 4. FTIR spectrum of cinnamaldehyde with attributions of the main bands.

S3. Determination of Surface Densities of APTES and CIN

S3.1. From TGA Analyses

The moles of APTES in 1 g of TO-APTES were obtained from the 3% weight loss starting at 280 °C in TG curves, according to the Equation (S1):

$$n_{APTES} = \frac{0.03\ g_{APTES}}{58\ g\ mol^{-1}} = 0.52\ mmol \quad \text{(S1)}$$

where 58 g/mol is the molecular weight of APTES alkyl chain (C_3H_8N). It should be noted that the ethoxide groups of APTES molecule were considered completely hydrolyzed on the basis of previous works [4] and on grounds of CHN analyses.

The mass of oxide present in 1 g of TO-APTES was calculated as follows:

$$m_{TiO_2} = 1 - \left(n_{APTES} \cdot 120\ g\ mol^{-1} + 0.01\ g_{H_2O}\right) = 0.93\ g \quad \text{(S2)}$$

where 120 g/mol is the molecular weight of the attached APTES ($C_3H_8NSiO_3$), assuming all the ethoxide groups were lost, and 0.01 g corresponds to the mass loss in the TG curve due to water loss.

Thus, by considering the specific surface area (SSA) of the adopted TiO_2 powder (50 m^2g^{-1}), the surface density of APTES, δ_{APTES}, in TO-APTES sample was calculated using Equation (S3):

$$\delta_{APTES} = \frac{n_{APTES} \cdot N_A}{m_{TiO_2} \cdot SSA_{TiO_2}} \cdot 10^{-18} \frac{m^2}{nm^2} = 6.9 \frac{molecules}{nm^2} \quad \text{(S3)}$$

where N_A is the Avogadro’s number (6.022 × 10^{23} molecules/mol).

From the TG analysis of TO-APTES-CIN, the surface coverage of both APTES and CIN were determined. In this sample, two main weight losses are observed, both accounting for 3%w: the first one could be attributed to the degradation of aliphatic chains and the second ascribable to the thermal decomposition of the aromatic ring of CIN. From the latter, the moles of grafted CIN in 1 g of TO-APTES-CIN were estimated according to the Equation (S4):

$$n_{CIN} = \frac{0.03\ g_{C_6H_5}}{77\ g\ mol^{-1}} = 0.39\ mmol \quad \text{(S4)}$$

where 77 g/mol corresponds to the molar mass of the aromatic ring.

The moles of APTES in 1 g of TO-APTES-CIN could then be determined from the initial weight loss, taking into account the alkyl chain in grafted CIN molecules:

$$n_{APTES} = \frac{0.03 g_{C_6H_9N} - (n_{CIN} \cdot 39\ \ g\ mol^{-1})}{58\ g\ mol^{-1}} = 0.26\ mmol \quad \text{(S5)}$$

where 39 and 58 g mol^{-1} are the molecular mass of the aliphatic moiety of grafted CIN (C_3H_3) and of the alkyl chain of covalently bonded APTES (C_3H_6N), respectively.

The mass of oxide in 1 g of TO-APTES-CIN was calculated as follows:

$$m_{TiO_2} = 1\ g - \left(n_{APTES} \cdot 120\ g\ mol^{-1} + n_{CIN} \cdot 116\ g\ mol^{-1} + 0.005\ g_{H_2O}\right) = 0.92\ g \quad \text{(S6)}$$

where 120 g/mol is the molecular weight of the attached APTES ($C_3H_8NSiO_3$), assuming all the ethoxide groups were lost, 116 g/mol is the molecular weight of the grafted CIN (C_9H_8) and 0.005 g corresponds to the mass loss in the TG curve due to water loss.

The surface densities of APTES moieties and CIN molecules in TO-APTES-CIN were calculated according to the Equation (S7) and Equation (S8):

$$\delta_{APTES} = \frac{n_{APTES} \cdot N_A}{m_{TiO_2} \cdot SSA_{TiO_2}} \cdot 10^{-18} \frac{m^2}{nm^2} = 3.5 \frac{molecules}{nm^2} \quad \text{(S7)}$$

$$\delta_{CIN} = \frac{n_{CIN} \cdot N_A}{m_{TiO_2} \cdot SSA_{TiO_2}} \cdot 10^{-18} \frac{m^2}{nm^2} = 5.1 \frac{molecules}{nm^2} \quad \text{(S8)}$$

S3.2. From CHN Analyses

The δ_{APTES} in TO-APTES was determined starting from the amount of N determined by CHN analyses (1.11%w, see Tab.1). The moles of APTES in 1 g of TO-APTES were determined according to the Equation (S9):

$$n_{APTES} = n_N = \frac{1.11 \cdot 10^{-2} g}{14\ g\ mol^{-1}} = 0.793\ mmol \quad (S9)$$

where 14 g/mol is the molar mass of N.

The weight percentage of Si within the sample was calculated as follows:

$$Si\% = n_{APTES} \cdot 28\ g\ mol^{-1} \cdot 100 = 2.22\%w \quad (S10)$$

where 28 g/mol is the molar mass of Si.

The total amount of oxide in 1 g of TO-APTES was determined as follows:

$$m_{TiO_2} = 1\ g - (3.23 \cdot 10^{-2} g + 1.30 \cdot 10^{-2} g + 1.11 \cdot 10^{-2} g + 2.22 \cdot 10^{-2} g) = 0.92\ g \quad (S11)$$

where $3.23 \cdot 10^{-2}\ g$, $1.30 \cdot 10^{-2} g$, $1.11 \cdot 10^{-2} g$, and $2.22 \cdot 10^{-2}\ g$ are, respectively, the masses of C, H, N and Si in 1 g of TO-APTES (Table 1)

The surface density of APTES moieties was thus determined as follows:

$$\delta_{APTES} = \frac{n_{APTES} \cdot N_A}{m_{TiO_2} \cdot SSA_{TiO_2}} \cdot 10^{-18} \frac{m^2}{nm^2} = 10.4 \frac{molecules}{nm^2} \quad (S12)$$

The surface densities of APTES and CIN in TO-APTES-CIN were also estimated from CHN analyses. The moles of APTES were determined from N content (0.43%w, Tab. 1) according to the Equation (S13):

$$n_{APTES} = n_N = \frac{0.43 \cdot 10^{-2} g}{14\ g\ mol^{-1}} = 0.307\ mmol \quad (S13)$$

The CIN amount was determined from C content (5.50%w, Tab.1), taking into account the fraction of carbon due to APTES molecules. In this respect, the ethoxide groups were considered fully hydrolyzed, as discussed in Section S3.1. The moles of CIN in 1 g of TO-APTES-CIN were calculated according to the Equation (S14):

$$n_{CIN} = \frac{n_{C_{tot}} - n_{C_{APTES}}}{9} = \left[\frac{5.50 \cdot 10^{-2}\ g}{12\ g\ mol^{-1}} - (3 \cdot n_{APTES})\right] \cdot \frac{1}{9} = 0.407\ mmol \quad (S14)$$

where 12 g/mol is the molar mass of carbon and 9 is the number of carbon atoms in a CIN molecule.

The weight percentage of Si within the sample was calculated as follows:

$$Si\% = n_{APTES} \cdot 28\ g\ mol^{-1} \cdot 100 = 0.86\%w \quad (S15)$$

The mass of the oxide in 1 g of TO-APTES-CIN was then calculated as follows:

$$m_{TiO_2} = 1g - (5.50 \cdot 10^{-2} g + 0.64 \cdot 10^{-2} g + 0.43 \cdot 10^{-2} g + 0.86 \cdot 10^{-2}\ g) = 0.93\ g \quad (S16)$$

where $5.50 \cdot 10^{-2}\ g$, $0.64 \cdot 10^{-2} g$, $0.43 \cdot 10^{-2} g$, and $0.86 \cdot 10^{-2}\ g$ are, respectively, the masses of C, H, N and Si in 1 g of TO-APTES-CIN (Table 1).

The surface densities of APTES moieties and CIN molecules in TO-APTES-CIN were calculated according to the Equation (S17) and Equation (S18):

$$\delta_{APTES} = \frac{n_{APTES} \cdot N_A}{m_{TiO_2} \cdot SSA_{TiO_2}} \cdot 10^{-18} \frac{m^2}{nm^2} = 4.0 \frac{molecules}{nm^2} \quad (S17)$$

$$\delta_{CIN} = \frac{n_{CIN} \cdot N_A}{m_{TiO_2} \cdot SSA_{TiO_2}} \cdot 10^{-18} \frac{m^2}{nm^2} = 5.3 \frac{molecules}{nm^2} \quad (S18)$$

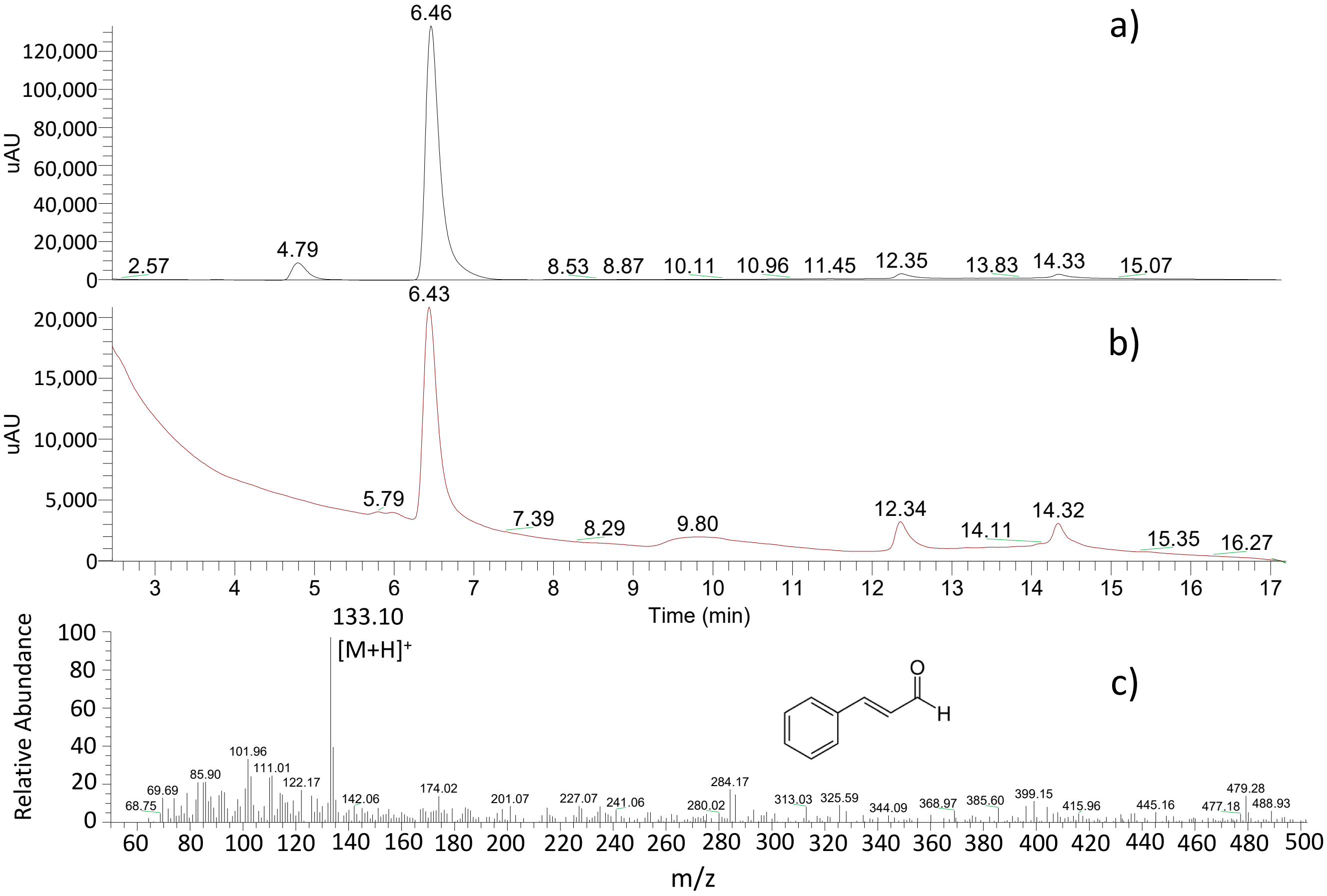

Figure S5. HPLC chromatograms of (**a**) reference CIN aqueous solution and (**b**) solution after release test of TO-APTES-CIN powder in ultrapure water at the end of the release test. (**c**) ESI-MS spectrum of the peak at R_T 6.4 min, with attribution of the main peak.

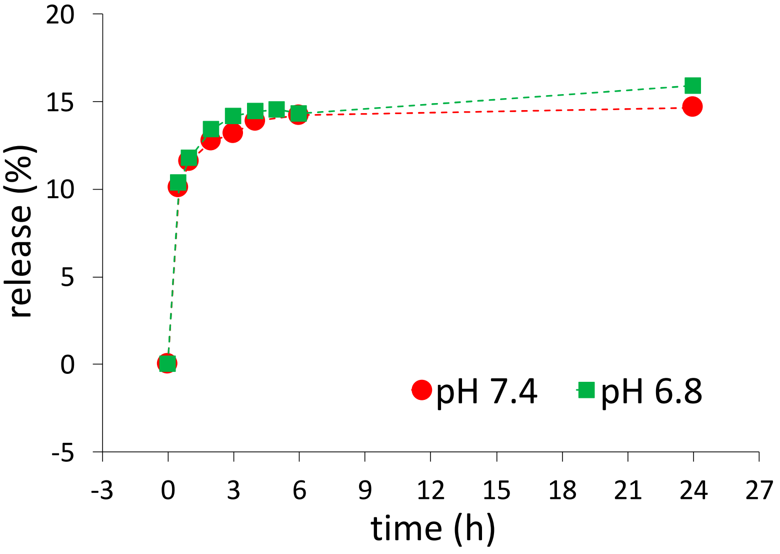

Figure S6. Cumulative release during tests at pH 6.8 and 7.4 for TO-APTES-CIN powders.

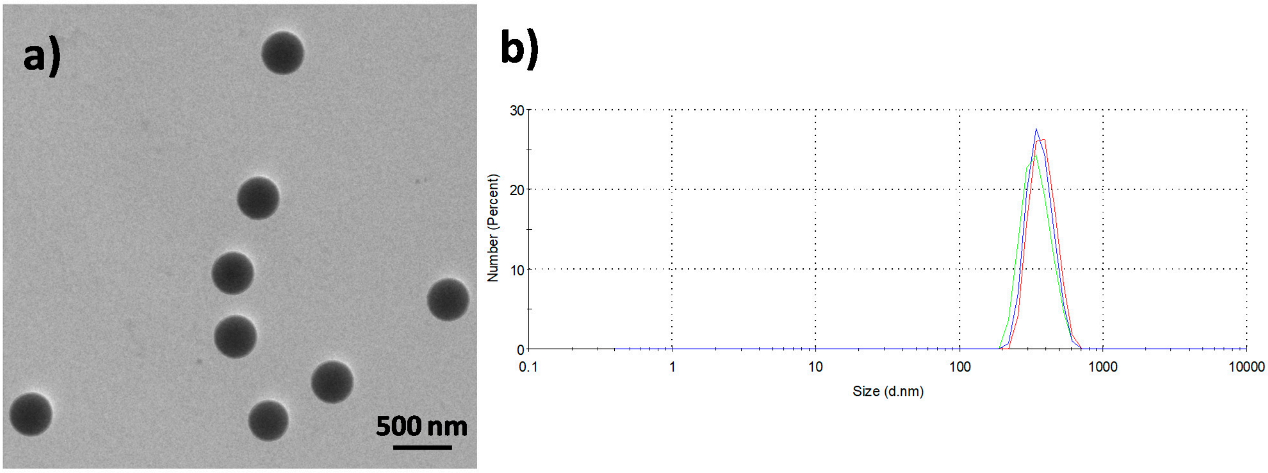

Figure S7. (**a**) TEM image of polystyrene NPs in water; (**b**) DLS size distribution by number of polystyrene NPs in ethylene glycol.

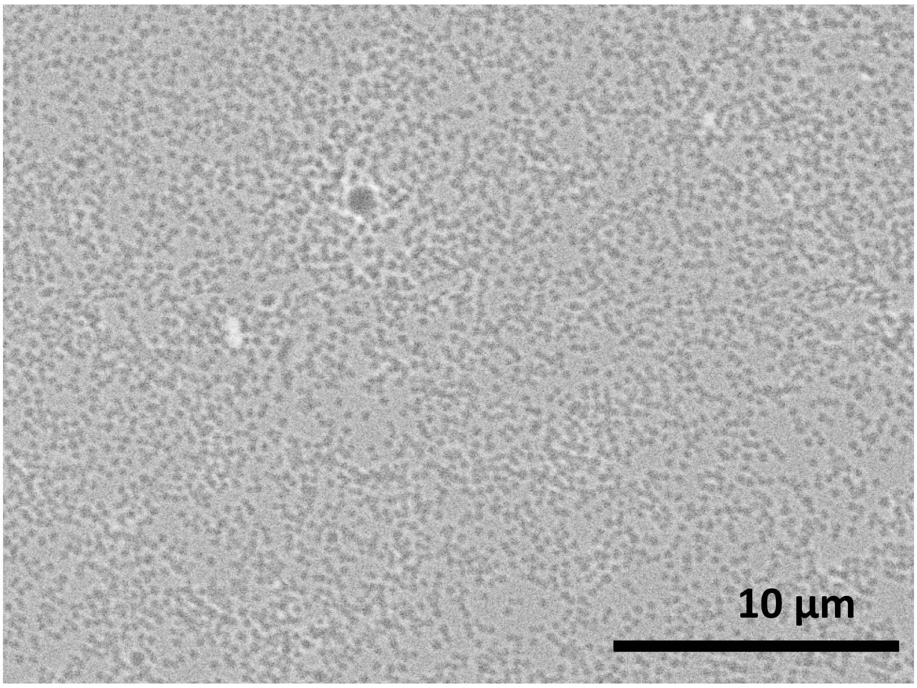

Figure S8. SEM image of the porous titania film.

References

1. Goodwin, J.W.; Hearn, J.; Ho, C.C. and Ottewill, R.H. Studies on the preparation and characterization of monodisperse polystyrene latices. *Colloid Polym. Sci.* **1974**, *252*, 464–47.
2. Steven E. Ruzin. *Plant microtechnique and microscopy*; Oxford University Press: Oxford, UK, 1999. doi:10.1046/j.1469-8137.2000.00735.
3. Steven E. Ruzin; Buffers. Available online: http://microscopy.berkeley.edu/Resources/instruction/buffers.html. (accessed on March 18, 2021)
4. Taroni, T. *et al.* Thiahelicene-grafted halloysite nanotubes: Characterization, biological studies and pH triggered release. *Appl. Surf. Sci.* **2020**, *520*, 146351.
